# Statistical analysis plan for the Surgery for Cancer with Option of Palliative Care Expert (SCOPE) trial: a randomized controlled trial of a specialist palliative care intervention for patients undergoing surgery for cancer

**DOI:** 10.1186/s13063-021-05256-y

**Published:** 2021-04-29

**Authors:** Onur M. Orun, Myrick C. Shinall, Aimee Hoskins, Ellis Morgan, Mohana Karlekar, Sara F. Martin, E. Wesley Ely, Rameela Raman

**Affiliations:** 1 Department of Biostatistics, Vanderbilt University Medical Center, 2525 West End, Suite 1100, Nashville, 37203 TN USA; 2 Critical Illness, Brain Dysfunction, and Survivorship Center, Vanderbilt University Medical Center, Nashville,, TN USA; 3 Division of General Surgery, Department of Surgery, Vanderbilt University Medical Center, Nashville,, TN USA; 4 Section of Palliative Care, Department of Medicine, Vanderbilt University Medical Center, Nashville,, TN USA; 5 Center for Biomedical Ethics and Society, Vanderbilt University Medical Center, Nashville,, TN USA; 6 Division of Allergy, Pulmonary, and Critical Care Medicine, Department of Medicine, Vanderbilt University Medical Center, Nashville,, TN USA; 7 Geriatrics Research, Education, and Clinical Center, Veterans Affairs Tennessee Valley Health System, Nashville,, TN USA

**Keywords:** Palliative care, Surgical oncology, Cancer, Randomized controlled trial, Statistical analysis plan

## Abstract

**Background:**

The impact of specialist palliative care intervention in patients undergoing surgery for cancer has not been studied extensively. The SCOPE randomized controlled trial will investigate the effect of specialist palliative care intervention in cancer patients undergoing surgery for selected abdominal malignancies. The study protocol of the SCOPE Trial was published in December 2019.

**Methods and design:**

The SCOPE Trial is a single-center, single-blind, prospective, randomized controlled trial that will investigate specialist palliative care intervention for cancer patients undergoing surgery for selected abdominal malignancies. The study plans to enroll 236 patients that will be randomized to specialist palliative care (intervention arm) and usual care (control arm) in a 1:1 ratio.

**Results:**

The primary outcome of the study is the Functional Assessment of Cancer Therapy-General (FACT-G) Trial Outcome Index (TOI) at 90 days postoperatively. Secondary outcomes of the study include the total FACT-G score at 90 days postoperatively, days alive at home without an emergency room visit within 90 days of operation, and all-cause mortality at 1 year after operation. Time frames for all outcomes will start on the day of surgery.

**Conclusion:**

This manuscript serves as the formal statistical analysis plan (version 1.0) for the SCOPE randomized controlled trial. The statistical analysis plan was completed on 6 April 2021.

**Trial registration:**

ClinicalTrials.gov NCT03436290. Registered on 16 February 2018

## Background

Palliative care is medical care focused on improving the quality of life of people with serious illnesses. The involvement of palliative care specialists has been shown to improve quality of life, reduce symptom burden, decrease healthcare utilization, and improve survival for patients with cancer managed medically [1–4]. However, little data exists on the benefits of specialist palliative care for patients undergoing surgery for cancer. The Surgery for Cancer with Option of Palliative Care Expert (SCOPE) Trial will assess the efficacy of a specialist palliative care intervention in patients undergoing surgical resections of abdominal malignancies.

This statistical analysis plan (SAP) was written following the guidelines for the statistical analysis plans by Gamble et al. [5].

### Study objectives

The SAP pertains to the following aims for the SCOPE randomized controlled trial:

1. To determine whether a preoperative, perioperative, and postoperative specialist palliative care consultation improves 90-day postoperative functional and physical quality of life of patients undergoing surgery for selected abdominal malignancies
2. To determine whether a preoperative, perioperative, and postoperative specialist palliative care consultation improves recovery after surgery for selected abdominal malignancies as reflected by overall quality of life at 90 days postoperatively, days alive at home without an emergency room visit in the first 90 postoperative days, and overall survival in the first postoperative year.

## Methods and design

### Trial design

The SCOPE Trial is a single-center, single-blind, prospective, randomized controlled trial. The intervention arm of the study includes patients receiving a preoperative outpatient specialty palliative care consultation (PCC) from a palliative care provider (physician or nurse practitioner) in addition to postoperative inpatient and outpatient palliative care follow-up. Control arm of the study includes patients receiving usual care with PCC available at the discretion of their clinical providers.

### Inclusion and exclusion criteria

Patients were included if they met all the inclusion criteria and none of the exclusion criteria as listed in the study protocol and published previously [6].

### Randomization

Patients were randomized to specialist palliative care (intervention arm) or usual care (control arm) in a 1:1 ratio using a computer-generated permuted block randomization scheme, stratified by cancer type. The primary biostatistician of the study created the randomization list, which was directly uploaded into REDCap’s randomization module [7, 8].

### Power and sample size

The minimal clinically important difference for the total FACT-G score is estimated to be between 3 and 7 points [9]. Another study has shown that early palliative care has an approximate effect size of 0.4 on TOI score [1]. To determine the sample size to achieve at least 80% power, we assumed a common standard deviation of 9 for the FACT-G TOI score and 18.1 for the total FACT-G score [9]. Thus, assuming a two-sided alpha of 0.05, 98 enrolled participants in each group (a total of 196 patients) would provide at least 80% power to detect at least 3.6 points difference in the FACT-G TOI score and 7.24 points in the total FACT-G score. To account for up to 20% loss to follow-up 90 days postoperatively, 118 patients in each group (a total of 236 patients) are planned to be enrolled in the study to have adequate power.

## Statistical principles

The statistical analyses for the aims specified will be conducted as per this SAP and follow the statistical principles described below. Long-term outcomes not specified in this SAP will be analyzed separately for a secondary manuscript.

### Descriptive statistics

Participant flow diagram of the SCOPE randomized controlled trial will be reported as specified by the Consolidated Standards of Reporting Trials (CONSORT) 2010 guidelines [10]. The flow diagram will display all stages of the trial such as screening, exclusions, enrollment, randomization, death, study withdrawal, and follow-up.

Demographic information (e.g., age, sex, race, among others), clinical characteristics at baseline (e.g., Charlson comorbidity index, cancer type, among others), and study outcomes will be presented for each study arm. Median and interquartile range (IQR) will be used for describing continuous variables, and frequency (percentage) will be used for describing categorical variables.

No hypothesis testing for the differences in baseline characteristics between the intervention arm and control arm will be conducted as recommended by the CONSORT 2010 guidelines for reporting parallel-group randomized clinical trials [10, 11].

### Confidence intervals and *P* values

Statistical significance level for all outcomes will be at the 0.05 (alpha = 0.05) level. 95% confidence intervals will be presented with all effect estimates. Clinical significance, effect estimates, and confidence intervals will be highlighted in the statistical analysis results.

### Modeling principles

Depending on the distribution of the variable, non-linear relationships between the outcome and the continuous covariates will be incorporated into the statistical models using restricted cubic splines (3–5 knots) when appropriate. To account for the correlation between study participants having the same cancer type, standard errors will be adjusted using the Huber-White (robust) sandwich estimator [12]. No multiple comparison adjustments will be made for secondary and exploratory outcomes following the standard practice of analyzing multiple, prospective clinical trial outcomes [13–16]. No subgroup analyses will be conducted. Statistical analysis results for secondary and exploratory outcomes will be interpreted cautiously.

### Missing data

Missing covariate data will be statistically imputed using model-based multiple imputation. For each statistical model, the outcome and all the covariates in the model will be included in the imputation model. If the percentage of the missing data is lower than 5%, single imputation will be used.

### Rigor, transparency, and reproducibility

All the statistical analyses for the study will be conducted as described in this formal statistical analysis plan. All statistical analysis code will be made publicly available after the main manuscript of the study is published.

### Adherence to the intervention and protocol deviations

Adherence to the intervention will be assessed based on the proportion of study participants in the intervention arm that did not receive PCC and the proportion of participants in the control arm that received PCC. Proportion of participants in the intervention arm that did not receive PCC, proportion of participants in the control arm that received PCC, proportion of participants that withdrew from the study, total number of inpatient and outpatient PCC received by the participants, and the days between surgery and the first PCC will be presented descriptively by the treatment group to assess the adherence to the intervention.

All deviations from the study protocol will be reported. Any deviations that cause safety risks to the study participants will also be considered as protocol deviations. The number of participants with protocol deviations will be reported descriptively by the treatment group.

## Statistical analysis

### Outcomes

Study outcomes and time frames are defined in Table 1. All time frames start at the date of operation (i.e., the day of operation is day 0, and the first postoperative day is day 1) or the date of scheduled operation for those that did not undergo surgery. Percentage of patients in the control arm who receive PCC will be reported descriptively.

**Table 1:** Primary, secondary, and exploratory outcomes

Variable	Description	Time frame
**Primary outcome**		
Physical and functional well-being measured by the FACT-G TOI	Sum of physical and functional well-being subscales of the FACT-G. Higher scores indicate better outcome.	90 days
**Secondary outcomes**		
Quality of life measured by the total FACT-G score	Total score of all subscales (physical well-being, social/family well-being, emotional well-being, functional well-being) of the FACT-G. Higher scores indicate better outcome.	90 days
Days alive at home without an emergency room visit	Days alive without an emergency room visit and without being an inpatient of a hospital or other healthcare facility.	90 days
All-cause mortality	Time to death (in days).	1 year
**Exploratory outcomes**		
Anxiety measured by PROMIS Anxiety *T*-score	*T*-score calculated from the PROMIS Emotional Distress-Anxiety 6a Short Form. Higher scores indicate more anxiety.	90 days
Depression measured by PROMIS Depression T-score	*T*-score calculated from the PROMIS Depression 6a Short Form. Higher scores indicate more symptoms of depression.	90 days
Time to initiation of adjuvant therapy	Time to initiation of adjuvant chemotherapy or radiation (in days). Death will be a competing risk.	90 days
Caregiver burden measured by the Short Form Zarit Burden Interview score	Short Form Zarit Burden Interview (ZBI-12) score. Higher scores indicate more caregiver burden.	90 days
Size of life space measured by the Life-Space Assessment Questionnaire	Life-Space Assessment Questionnaire score. Higher scores indicate larger life space.	90 days

*FACT-G* Functional Assessment of Cancer Therapy-General, *TOI* Trial Outcome Index, *PROMIS* Patient-Reported Outcomes Measurement Information System

### Analysis population

Analysis population for all outcomes will be based on the intent-to-treat (ITT) principle. Complete-case analysis will be conducted for all 90-day assessment outcomes. We anticipate mortality and loss to follow-up to be very low 90 days postoperatively and do not expect it to be associated with group assignment. Thus, conducting a complete-case analysis for 90-day assessments is likely to provide an unbiased estimate of the effect of treatment on the outcome [17]. Sensitivity analyses as in the “Sensitivity analysis” section will be conducted to evaluate the robustness of our results to these assumptions.

### Analysis methods and model assumptions

The primary, secondary, and exploratory outcomes listed in Table 1 will be analyzed using unadjusted analyses as well as multivariable regression methods, adjusting for covariates listed in the “Model covariates” section. Theoretically, baseline measures of the study participants should be balanced between the intervention arm and the control arm due to randomization. However, adjustment increases study power as well as the precision of the study [18, 19]. Thus, adjusted analyses will be considered as the primary analyses unless issues of model convergence arise as mentioned in the“Model covariates” section.

Proportional odds logistic regression will be used for modeling continuous outcomes that are non-normally distributed [20, 21]. Proportional odds assumptions will be checked graphically using multiple cutoffs of the outcome, i.e., binary logistic regression models will be fitted using different cutoffs of the outcome, and the log odds ratios (estimated coefficients) will be plotted against cutoffs to check proportionality assumption [20]. Odds ratios and adjusted medians by treatment group along with 95% confidence intervals will be reported for these models.

Multivariable linear regression will be used for continuous outcomes that are normally distributed and satisfy the other assumptions. Assumptions will be checked graphically using residuals versus fitted values plots and quantile-quantile plots. Regression coefficients along with 95% confidence intervals will be reported for multivariable linear regression.

For 1-year all-cause mortality, Cox proportional hazards regression will be used along with Kaplan-Meier survival curve. Cox proportional hazards regression assumptions will be checked graphically using Schoenfield residuals versus time plots. Hazard ratios along with 95% confidence intervals will be reported for the Cox proportional hazards model. Patients alive at 1 year will be censored at 1 year (365 days). Patients whose death information is unknown will be censored at their last known date alive.

To analyze time to initiation of adjuvant therapy outcome, cumulative incidence curves will be generated using death as competing risk. Fine-Gray competing risk regression will be used for adjusted analysis.

Unadjusted analyses for all outcomes will be conducted without any covariates in the models except for the treatment group.

### Model covariates

All outcomes except 1-year all-cause mortality and time to initiation of adjuvant therapy within 90 days will be adjusted for using the following covariates:

- Baseline measure of the follow-up outcome (when available)
- Type of cancer (categorical variable with four levels: foregut carcinoma, colorectal carcinoma, bladder cancer, all other cancer types)
- Baseline frailty measured by risk analysis index (RAI) score (continuous)
- Age at randomization in years (continuous)
- Sex (categorical variable with two levels: female, male)

One-year all-cause mortality and time to initiation of adjuvant therapy within 90 days (starting at the time of operation or scheduled operation for those that did not undergo surgery) will be adjusted for using the following covariates:

- Type of cancer (categorical variable with four levels: foregut carcinoma, colorectal carcinoma, bladder cancer, all other cancer types)
- Baseline frailty measured by RAI score (continuous)
- Age at randomization in years (continuous)
- Sex (categorical variable with two levels: female, male)

Redundancy analysis will be performed before modeling using an adjusted *R*^2^ cutoff of 0.7 to evaluate for multicollinearity. Covariates that are highly correlated will be dropped from the model based on the rank order specified above.

If the model is overfit, covariates will be reduced based on the rank order listed above. If the counts in each level of the variable, type of cancer, is too low and cause convergence issues, we will reduce it to two levels. For every 15 events, 1 degree of freedom will be spent in the model. If there are not enough events to include any covariates for the 1-year mortality outcome, the Kaplan-Meier survival curve stratified by group will be presented. If there are not enough events to include any covariates for the time to initiation of adjuvant therapy outcome, only the cumulative incidence curve by group will be presented.

### Sensitivity analysis

Differential follow-up in the two study arms will be examined to evaluate for attrition bias. The Kaplan-Meier curve will be presented for time to dropout (including death) for the two study arms. If proportion of patients that were assessed at 90 days for the primary outcome between the two treatment groups is statistically significant at 5%, a sensitivity analysis will be conducted for the primary outcome, FACT-G TOI, and the secondary outcome, total FACT-G score, where the follow-up measure will be imputed by carrying the last observation (i.e., baseline observation) forward.

### Safety data

Due to the low-risk nature of the intervention, participation in the study or receiving PCC are not expected to have any adverse events (AEs) [6]. Detailed information about safety monitoring were included in the protocol [6]. If any AEs (e.g., excessive anxiety due to quality of life follow-up assessments) occur, all the AEs will be reported descriptively by the treatment group.

## Software details

R version 4.0.3 (or above) will be used for all analyses [22].

## Conclusion

This manuscript serves as the formal statistical analysis plan (version 1.0; 6 April 2021) for the SCOPE Trial. All statistical analyses will be conducted as specified in the statistical analysis plan. Amendments (if any) to this SAP will be reported.

## Trial status

Recruitment status: Enrolling by invitationProtocol version: 1.06 (1 February 2021)Recruitment start date: 1 March 2018 Approximate recruitment completion date: 1 December 2021

## Data Availability

Not applicable. Declarations

## Abbreviations

AEs: Adverse events
CONSORT: Consolidated Standards of Reporting Trials
FACT-G: Functional Assessment of Cancer Therapy-General
IQR: Interquartile range
IRB: Institutional review board
ITT: Intent-to-treat
PCC: Palliative care consultation
PROMIS: Patient-Reported Outcomes Measurement Information System
RAI: Risk analysis index
SAP: Statistical analysis plan
TOI: Trial Outcome Index
SCOPE: Surgery for Cancer with Option for Palliative Care Expert
ZBI: Zarit Burden Interview
